# Relationship between sleep and obesity among U.S. and South Korean college students

**DOI:** 10.1186/s12889-020-8182-2

**Published:** 2020-01-22

**Authors:** Jaesin Sa, Siyoung Choe, Beom-young Cho, Jean-Philippe Chaput, Gyurin Kim, Chae-Hee Park, Joon Chung, Yoojin Choi, Beatrice Nelson, Yongkyu Kim

**Affiliations:** 1 College of Education and Health Sciences, Touro University, Vallejo, CA USA; 2 Department of Kinesiology and Health, Miami University, Oxford, OH USA; 3 Department of Epidemiology, University of Michigan, Ann Arbor, MI USA; 4 Department of Pediatrics, University of Ottawa, Ottawa, ON Canada; 5 Department of Physical Education, Seoul National University, Seoul, South Korea; 6 Department of Sport and Healthy Aging, Korea National Sport University, Seoul, South Korea; 7 Division of Sleep and Circadian Disorders, Harvard Medical School, Boston, MA USA; 8 Department of Secondary and Physical Education Salisbury University, Salisbury, MD USA; 9 Department of Kinesiology, University of Maryland Eastern Shore, Princess Anne, MD USA; 10 Department of Physical Education, Daegu University, Daegu, South Korea

**Keywords:** Sleep, Obesity, Race, South Korea, Gender

## Abstract

**Background:**

Little is known about the relationship between sleep and obesity in young adults, particularly college students. This study examined the relationship between sleep (i.e., sleep duration and quality) and obesity in a large and diverse binational sample of college students.

**Methods:**

Analyses were based on a 40-item paper survey from 2016/2017 to 2017/2018 academic years, with a 72% response rate. The samples were 1578 college students aged 18–25 years from five universities (two in the U.S. and three in South Korea). Weight and height were measured objectively; other measures (e.g., health behaviors) were self-reported. Multinomial logistic regression was used to assess the association between sleep duration and independent variables (race/nationality, gender, and BMI). Poisson regression was used to examine the relationship between sleep quality and independent variables.

**Results:**

Overall, blacks had a higher adjusted odds ratio (AOR) of short sleep (< 7 h/night) than whites (AOR = 1.74, *P* < .01); overweight participants had a higher AOR of short sleep than normal weight participants (AOR = 1.52, *P* < .01); and obese participants had a higher AORs of both short and long sleep (> 9 h/night) (AOR = 1.67, *P* < .01; AOR = 1.79, *P* < .05, respectively). Among men, being black, overweight, and obesity were associated with short sleep (*P* < .05), whereas only obesity was related to short sleep among women (*P* < .05). In analyses stratified by race and nationality, overweight and obesity were related to short sleep among blacks only (*P* < .05). Overall, sleep quality (getting enough sleep to feel rested in the morning in the past 7 days) was worse in blacks and South Koreans than whites (*P* < .05), worse in women than men (*P* < .05), and worse in participants with obesity than normal weight participants (*P* < .05).

**Conclusions:**

Obesity was associated with both short (< 7 h/night) and long sleep duration (> 9 h/night) and poor sleep quality among all participants. In comparison with whites, blacks were more like to have short sleep, and blacks and South Koreans had worse sleep quality. Further investigations using a larger sample of college students in multiple countries may be helpful to identify target populations who are at a greater risk of obesity and sleep problems.

## Background

Sleep problems are considered an emerging global epidemic [1, 2]. In the United States (U.S.), sleep problems are estimated to affect 50–70 million adults every year [3]. Sleep problems are associated with multiple disabilities, morbidity, mortality, depression [4], and unhealthy behaviors [3, 5] including cigarette use [6], alcohol use [7], lower intakes of fruit and vegetable [8], and lack of physical activity [9]. College students are at high risk of sleep problems [2, 10], as they not only have high likelihood of sleep problems, but are also likely to develop long term problems associated with chronic sleep deprivation [11, 12].

Studies have reported that trends in sleep problems parallel trends in obesity [1, 13]. Obesity is another global epidemic that affects approximately 5.5% of current college students across different geographic regions, with notable national disparities [14]. Sleep problems contribute to obesity, as shorter duration and poorer quality of sleep lead to behavioral, metabolic, and endocrine changes that lead to weight gain [15, 16]. At the same time, obesity contributes to sleep problems, as individuals with obesity are more likely to suffer from sleep disordered breathing, such as sleep apnea [17]. Despite the parallels and the mechanism that drive those parallels, the relationship between sleep problems and obesity among college students is not fully understood [16, 18]. Our understanding is particularly limited for gender and racial differences in sleep and obesity among college students.

Studies have reported that people manifest problematic sleep in different ways. For instance, studies have suggested significant gender differences in risk or prevalence of both obesity and sleep problems [3, 19]. The National Institute of Health (NIH) specifically mentioned the identification of gender differences that contribute to the risk of sleep problems as one of their five goals of their 2011 Sleep Disorders Research Plan [3]. One study found that the relationship between sleep duration and body mass index (BMI) is affected by gender instead of race [20], though it is unclear if the same is true for the relationship between sleep quality and BMI.

Nevertheless, sleep problems and obesity are affected by race. Non-Hispanic blacks (blacks) are more likely to report short sleep durations [21], while having higher BMI than non-Hispanic whites (whites) [22], suggesting that sleep problems have greater influence on obesity status for certain racial groups [23, 24]. However, many existing studies conflate various racial groups, limiting inference therein. Many of the existing studies either do not include non-Hispanic Asians (Asians) in their study sample, or include Asians as one combined racial group [23]. Differences in ethnicity, nativity, and primary language are associated with different health and behaviors within the Asian population [25], and Asians with different nationalities should be considered separately.

South Korea (Korea) is a country with a high prevalence of sleep problems [26, 27], with higher prevalence of insomnia (22.8%) than other Asian countries (e.g., Singapore: 15.3% and Japan: 21.4%) [27–29]. Korea is also a country with a steady increase in the prevalence of obesity and severe obesity among adults since 1998 [30]. However, the health of college students is often overlooked [31, 32], and neither obesity nor sleep problems are rigorously monitored in the Korean college student population. To the best of our knowledge, there are no studies that have examined the relationship between sleep and obesity among Korean college students.

Only a few studies have examined the relationship between sleep and obesity in U.S. college populations, and even fewer studies included both duration and quality measures of sleep while considering demographic variance of the relationship [12, 18, 33, 34]. Moreover, none of the studies to date have investigated differences in sleep and obesity between college students in the U.S. and college students in Korea. The purpose of this study was thus to examine the relationship between sleep (i.e., sleep duration and quality) and body weight in a binational sample of college students, and identify gender and racial/national groups most affected by the relationship.

## Methods

### Survey and study sample

A 40-item paper questionnaire in English was adapted from the National Health and Nutrition Examination Survey (NHANES), the American College Health Association-National College Health Assessment (ACHA-NCHA), and the Center for Epidemiologic Studies Depression Scale (CES-D). NHANES collects the nation’s primary source of health-related data [35, 36], and ACHA-NCHA [37] and CES-D [38] have been determined to be of high reliability and high validity. The original 40-item English questionnaire was translated into Korean by two bilingual health professors at two universities in Korea, using translation steps to ensure that the translated questionnaire is valid and reliable [39]. A back translation was performed by two bilingual health professors in the U.S. Then, the jury of bilingual experts in the U.S. and Korea reviewed the original and translated questionnaires and edited the translated version to remove discrepancies. The translated questionnaire was pilot tested with ten undergraduates at participating universities in Korea to evaluate the questionnaire wording and respondent’s understanding of the questions. No wording problems were found, and this pilot sample was not included in the study sample. The current study was approved by the Institutional Review Board at five participating universities in the U.S. and Korea.

Study participants were undergraduates aged 18 to 25 years at a predominantly non-Hispanic white university, a historically black university, and three universities in Korea utilizing data from academic years 2016–2017 and 2017–2018. Convenience sampling was used for this study. A total of 2232 undergraduates enrolled in health courses (e.g., Foundations in Physical Education) were invited to participate in the 40-item paper survey by an email invitation that was sent by the five health departments at the five participating universities. Lead investigators at each university provided students with an overview of the study during class sessions. Inclusion criteria were being blacks, whites, Koreans, and aged 18 to 25 years. An exclusion criterion included pregnant students. A total of 1605 students participated (72% response rate), and 27 questionnaires with 10% or more missing responses were excluded based on NHANES analytic guidelines [40]. The final sample size was reduced to 1578 participants (60.3% Koreans) on five campuses.

### Measures

Race/nationality was self-reported and included non-Hispanic whites, non-Hispanic blacks, and non-Hispanic Asians. Annual family income was measured by an open-ended question “*What is the total income received last year by you and your family members before taxes*? *Family is individuals and groups of individuals who are related by birth, marriage or adoption*.” Using a Detecto scale with height bar (Detecto Scale Company, Webb City, MO), weight and height of participants in the U.S. and Korea were measured while wearing light clothing without shoes. Detecto is the largest medical scale manufacturer in the world [41] and is used as height and weight measurements in obesity studies [42, 43]. Weight and height were measured twice, and the mean of each measurement was recorded by lead investigators at each university. BMI was calculated as kg/m^2^ using participants’ measured height in centimeters and weight in kilograms. Standard BMI cut points (underweight = BMI < 18.5; normal = 18.5 ≤ BMI < 25.0; overweight = 25.0 ≤ BMI < 30.0; and obese = BMI ≥ 30.0) [44] and Asian BMI cut points (underweight = BMI < 18.5; normal = 18.5 ≤ BMI < 23.0; overweight = 23.0 ≤ BMI < 25.0; and obese = BMI ≥ 25.0) [45, 46] were used for U.S. students and Korean students, respectively.

General health was assessed with the question, “*How would you describe your general health*?” with response options of excellent, very good, good, fair, and poor. Participants answered dichotomous questions regarding sleep problems: (1) “*Have you ever told a doctor or other health professional that you have trouble sleeping?*” and (2) “*Have you ever been told by a doctor or other health professional that you have a sleep disorder?*” Sleep duration was measured by an open-ended question “*How much sleep do you usually get at night on weekdays or workdays?*” Responses to this question were grouped into short sleep (< 7 h/night), normal sleep (7–9 h/night), and long sleep (> 9 h/night) [47]. Sleep quality was assessed with the question, “*On how many of the past 7 days did you get enough sleep so that you felt rested when you woke up in the morning?*” Response options ranged from 0 days to 7 days.

Depression was measured by the CES-D scale, a 20-item self-report measure with response options ranging from 0 to 3 for each item (0 = rarely or none of the time, 1 = some or little of the time, 2 = moderately or much of the time, 3 = most or almost all the time). CES-D scores of 16 or higher is considered depressed.

Based on the Centers for Disease Control and Prevention glossary [48], current smoking (i.e., ever smoked 100 cigarettes in entire life and reported current cigarette smoking every day or some days) was measured by (1) “*Have you smoked 100 cigarettes in your entire life?*” and (2) “*Do you now smoke cigarettes every day, some days, or not at all?*” Alcoho use was assessed by an open-ended question “*Within the last 30 days, on how many days did you use alcohol (beer, wine, liquor)?*” Daily fruits and vegetables intake was measured with an open-ended question “*How many servings of fruits and vegetables do you usually have per day (1 serving = 1 medium piece of fruit; 1/2 cup fresh, frozen, or canned fruits/vegetables; 3/4 cup fruit/vegetable juice; 1 cup salad greens; or 1/4 cup dried fruit)?*”

Weekly moderate-intensity exercise for ≥10 min and weekly vigorous-intensity exercise for ≥10 min were assessed by open-ended questions (1) “*In a typical week, on how many days do you do moderate-intensity sports, fitness or recreational activities? Moderate-intensity sports, fitness or recreational activities cause small increases in breathing or heart rate such as brisk walking, bicycling, swimming, or golf for at least 10 minutes continuously*.” and (2) “*In a typical week, on how many days do you do vigorous-intensity sports, fitness or recreational activities? Vigorous-intensity activity causes large increases in breathing or heart rate like running or basketball for at least 10 minutes continuously*.”

### Statistical analysis

Chi-square tests were performed to identify significant differences in demographics and health behaviors among the three racial/national groups (whites, blacks, and Koreans). Data were analyzed separately for each gender and racial/national group. Racial/national differences in sleep quality were examined by using ANOVA followed by the Scheffe post hoc test. Multinomial logistic regression models were used to compute adjusted odds ratios (AORs) and investigate the association between sleep duration and three independent variables (race/nationality, gender, and BMI) with adjustment for potential confounding factors (age, annual family income, days of vigorous-intensity activity, days of moderate-intensity activity, current smoking status, days of alcohol use, having trouble sleeping, sleep disorder, and depression) based on prior research [49–51]. Whites were used as the reference group for all multinomial regression models. The prevalence of obesity by sleep duration was illustrated separately for race/nationality in graphical plots. Poisson regression models were used to estimate adjusted incidence rate ratio and examine the relationship between sleep quality (getting enough sleep to feel rested in the morning in the past 7 days) and three independent variables (race/nationality, gender, and BMI) with adjustment for confounding variables (age, current smoking status, days of alcohol use, and depression) based on previous studies [18, 49, 52]. All analyses were conducted with STATA version 13 (STATA Press, College Station, TX).

## Results

The study sample consisted of 1578 participants, with a mean age of 21.0 years (SD = 2.3). The majority of the study sample was comprised of men (61.3%), with 19.4% whites, 20.3% blacks, and 60.3% Koreans. The majority of men and women perceived their general health as good or very good. Among men, Koreans had higher overweight/obesity rates (59.4%) than blacks (51.5%) and whites (46.8%) when the World Health Organization (WHO) definition of overweight/obesity for Asian adults was used. However, Koreans had lower overweight/obesity rates (26.2%) when the same BMI criteria were used across the three racial/national groups.

As shown in Table 1, more white men had normal sleep duration (*P* < .001), better sleep quality (i.e., getting enough sleep to feel rested in the morning in the past 7 days) (*P* < .05), and more moderate-intensity exercise (*P* < .001) compared to black or Korean men. Korean men had less trouble sleeping (*P* < .001) and had higher CES-D scores (*P* < .01) compared to white or black men. Korean men had the highest current smoking rate (30.9%) compared to white (13.4%) or black men (2.1%) (*P* < .001) and white men had the highest fruit and vegetable consumption (*P* < .001).

**Table 1:** Characteristics of men by race/nationality (*n* = 968)

Demographics and Health Behaviors	Whites (*n* = 188)	Blacks (*n* = 196)	Koreans (*n* = 584)	*p* value
	%	%	%	
Years in undergraduate school				<.001
1 year	12.8	41.0	28.9	
2 years	12.2	24.1	25.7	
3 years	24.5	16.9	25.0	
4 years	33.0	11.3	17.6	
≥ 5 years	17.6	6.7	2.7	
General health				<.001
Excellent	17.1	13.4	13.0	
Very good	45.5	38.1	30.9	
Good	31.6	38.1	31.4	
Fair	5.9	10.3	24.7	
Poor	0.5	1.0	4.8	
Body mass index^a^				.003
Underweight	1.1	1.0	1.0	
Normal weight	52.1	47.5	39.6	
Overweight	33.0	35.7	33.2	
Obesity	13.8	15.8	26.2	
Sleep duration^b^				<.001
Short sleep (< 7 h/night)	39.0	59.2	46.6	
Normal sleep (7–9 h/night)	50.3	35.7	47.6	
Long sleep (> 9 h/night)	10.7	5.1	5.8	
Having trouble sleeping	14.4	13.3	4.8	<.001
Having a sleep disorder	3.2	4.6	2.1	.163
Depression	23.5	27.0	34.9	.005
Current smoker	13.4	2.1	30.9	<.001
Alcohol use in last 30 days				<.001
0 days	10.6	51.5	8.2	
1–9 days	47.3	39.8	55.8	
≥ 10 days	42.0	8.7	36.0	
Daily fruits and vegetables intake				<.001
0 servings	3.2	10.2	15.9	
1–2 servings	73.9	60.7	70.7	
≥ 3 servings	22.9	29.1	13.4	
Weekly moderate-intensity exercise for ≥10 min				<.001
0 days	1.1	14.3	12.8	
1–2 days	26.6	28.1	25.7	
≥ 3 days	72.3	57.7	61.5	
Weekly vigorous-intensity exercise for ≥10 min				.030
0 days	5.3	12.8	9.8	
1–2 days	23.9	28.1	30.3	
≥ 3 days	70.7	59.2	59.9	
	Mean (SD)	Mean (SD)	Mean (SD)	
Days of getting enough sleep to feel rested in the morning in past 7 days	3.58 (2.11)^b^	2.99 (2.39)^c^	2.71 (1.78)^c,d^	<.05

The percentages may not add up to 100% because of no responses or rounding errors

Only one category for dichotomous variables is presented to eliminate redundancy in the table

^a^Underweight (BMI < 18.5), normal (18.5 ≤ BMI < 25.0), overweight (25.0 ≤ BMI < 30.0), and obese (BMI ≥ 30.0) for whites and blacks. Underweight (BMI < 18.5), normal (18.5 ≤ BMI < 23.0), overweight (23.0 ≤ BMI < 25.0), and obese (BMI ≥ 25.0) for Koreans

*P* values are based on a chi-square test for categorical variables and an ANOVA for continuous data. ^b-d^ Values with different superscripts were significantly different across racial/national groups in the Scheffe test after ANOVA

As shown in Table 2, the majority of women perceived their general health as good or very good. Black women had higher overweight/obesity (53.8%) compared to white (38.8%) or Korean women (24.8%) when using the WHO definition. Overweight/obesity rates (12.2%) were however lower for Korean women when the same BMI criteria were used across the three racial/national groups. Korean women had worse sleep quality (*P* < .05), had less trouble sleeping (*P* < .001), and had higher CES-D scores (*P* < .05) compared to white or black women. White women had more days of alcohol use (*P* < .001), had the highest fruit and vegetable consumption (*P* < .01), and did more vigorous-intensity exercise compared to blacks or Koreans (*P* < .001).

**Table 2:** Characteristics of women by race/nationality (*n* = 610)

Demographics and Health Behaviors	Whites (*n* = 98)	Blacks (*n* = 290)	Koreans (*n* = 222)	*p* value
	%	%	%	
Years in undergraduate school				<.001
1 year	6.1	43.1	21.8	
2 years	6.1	27.8	30.0	
3 years	33.7	17.0	26.4	
4 years	40.8	9.0	19.6	
≥ 5 years	13.3	3.1	2.3	
General health				<.001
Excellent	8.2	4.5	5.4	
Very good	37.8	22.5	21.6	
Good	43.9	47.1	30.2	
Fair	9.2	21.1	35.6	
Poor	1.0	4.8	7.2	
Body mass index^a^				<.001
Underweight	1.0	1.4	8.6	
Normal weight	60.2	44.8	66.7	
Overweight	25.5	25.5	12.6	
Obesity	13.3	28.3	12.2	
Sleep duration^b^				.450
Short sleep (< 7 h/night)	53.1	59.3	51.8	
Normal sleep (7–9 h/night)	38.8	34.8	41.9	
Long sleep (> 9 h/night)	8.2	5.9	6.3	
Having trouble sleeping	25.5	19.3	6.8	<.001
Having a sleep disorder	1.0	5.5	3.6	.136
Depression	42.7	41.7	52.7	.038
Current smoker	2.0	1.7	4.6	.140
Alcohol use in last 30 days				<.001
0 days	12.2	42.4	14.0	
1–9 days	54.1	49.0	60.4	
≥ 10 days	33.7	8.6	25.7	
Daily fruits and vegetables intake				.002
0 servings	5.1	10.3	13.5	
1–2 servings	58.2	66.9	68.9	
≥ 3 servings	36.7	22.8	17.6	
Weekly moderate-intensity exercise for ≥10 min				.060
0 days	12.2	25.9	25.7	
1–2 days	35.7	30.3	27.5	
≥ 3 days	52.0	43.8	46.9	
Weekly vigorous-intensity exercise for ≥10 min				<.001
0 days	22.5	41.7	39.2	
1–2 days	22.5	28.6	28.4	
≥ 3 days	55.1	29.7	32.4	
	Mean (SD)	Mean (SD)	Mean (SD)	*p* value
Days of getting enough sleep to feel rested in the morning in past 7 days	2.80 (2.17)^b,c^	2.83 (2.09)^b^	2.28 (1.63)^c^	<.05

The percentages may not add up to 100% because of no responses or rounding errors

Only one category for dichotomous variables is presented to eliminate redundancy in the table

^a^Underweight (BMI < 18.5), normal (18.5 ≤ BMI < 25.0), overweight (25.0 ≤ BMI < 30.0), and obese (BMI ≥ 30.0) for whites and blacks. Underweight (BMI < 18.5), normal (18.5 ≤ BMI < 23.0), overweight (23.0 ≤ BMI < 25.0), and obese (BMI ≥ 25.0) for Koreans

*P* values are based on a chi-square test for categorical variables and an ANOVA for continuous data. ^b-c^ Values with different superscripts were significantly different across racial/national groups in the Scheffe test after ANOVA

Table 3 summarizes the influence of gender, race/nationality, and BMI on sleep duration by gender and race/nationality, adjusting for demographic and health characteristics (age, annual family income, days of vigorous-intensity activity, days of moderate-intensity activity, current smoking status, days of alcohol use, having trouble sleeping, sleep disorder, and depression). Among all participants, blacks had a higher AOR of short sleep than whites (*P* < .01). Overweight participants had a higher AOR of short sleep than those who were normal weight (*P* < .01), while obese participants had a higher AOR of both short sleep (*P* < .01) and long sleep (*P* < .05).

**Table 3:** Multinomial logistic regression of sleep duration (*N* = 1578)

	All		Men		Women	
	Short sleep AOR (95% CI)^a^	Long sleep AOR (95% CI)^a^	Short sleep AOR (95% CI)^a^	Long sleep AOR (95% CI)^a^	Short sleep AOR (95% CI)^a^	Long sleep AOR (95% CI)^a^
Race/nationality						
Whites	Reference	Reference	Reference	Reference	Reference	Reference
Blacks	1.74 (1.20, 2.51)^**^	0.85 (0.43, 1.69)	2.22 (1.34, 3.68)^**^	0.93 (0.35, 2.46)	1.28 (0.70, 2.35)	0.59 (0.19, 1.84)
Koreans	0.97 (0.69, 1.36)	0.78 (0.43, 1.41)	1.05 (0.68, 1.61)	0.79 (0.38, 1.65)	0.91 (0.50, 1.68)	0.52 (0.16, 1.65)
BMI						
Underweight	0.86 (0.39, 1.93)	1.44 (0.39, 5.30)	0.69 (0.16, 2.94)	0.25 (0.17, 3.95)	1.00 (0.36, 2.77)	2.40 (0.56, 10.36)
Normal weight	Reference	Reference	Reference	Reference	Reference	Reference
Overweight	1.52 (1.16, 1.99)^**^	1.17 (0.67, 2.03)	1.48 (1.06, 2.07)^*^	1.10 (0.56, 2.19)	1.58 (0.97, 2.56)	1.42 (0.53, 3.78)
Obesity	1.67 (1.16, 2.41)^**^	1.79 (1.03, 3.43)^*^	1.61 (1.04, 2.74)^*^	1.68 (0.69, 4.09)	1.84 (1.07, 3.16)^*^	1.74 (0.62, 4.86)
	Whites		Blacks		Koreans	
	Short sleep AOR (95% CI)^a^	Long sleep AOR (95% CI)^a^	Short sleep AOR (95% CI)^a^	Long sleep AOR (95% CI)^a^	Short sleep AOR (95% CI)^a^	Long sleep AOR (95% CI)^a^
Gender						
Men	Reference	Reference	Reference	Reference	Reference	Reference
Women	1.36 (0.72, 2.56)	1.20 (0.42, 3.44)	0.95 (0.61, 1.47)	1.12 (0.44, 2.83)	1.08 (0.72, 1.63)	0.80 (0.34, 1.90)
BMI						
Underweight	0.82 (0.04, 18.08)	1.45 (0.91, 4.27)	0.22 (0.02, 2.09)	3.14 (0.28, 34.53)	1.19 (0.44, 3.19)	1.40 (0.26, 7.46)
Normal weight	Reference	Reference	Reference	Reference	Reference	Reference
Overweight	1.51 (0.77, 2.97)	1.73 (0.60, 5.02)	1.70 (1.06, 2.72)^*^	1.30 (0.43, 3.92)	1.30 (0.86, 1.97)	0.73 (0.28, 1.89)
Obesity	1.67 (0.71, 3.92)	2.63 (0.71, 9.77)	1.68 (1.01, 2.84)^*^	2.25 (0.80, 6.27)	1.10 (0.43, 2.77)	0.46 (0.05, 4.00)

Sleep duration classified into short sleep (< 7 h/night), normal sleep (7–9 h/night), and long sleep (> 9 h/night)

Underweight (BMI < 18.5), normal weight (18.5 ≤ BMI < 25.0), overweight (25.0 ≤ BMI < 30.0), and obesity (BMI ≥ 30.0) for whites and blacks. Underweight (BMI < 18.5), normal weight (18.5 ≤ BMI < 23.0), overweight (23.0 ≤ BMI < 25.0), and obesity (BMI ≥ 25.0) for Koreans

^a^Odds ratio adjusted for age, annual family income, days of vigorous-intensity activity, days of moderate-intensity activity, current smoking status, days of alcohol use, having trouble sleeping, sleep disorder, and depression

*AOR* adjusted odds ratio, *CI* confidence interval

^*^p < .05; ^**^p < .01

Among men, being black (*P* < .01), overweight (*P* < .05), and obesity (*P* < .05) were associated with short sleep. Among women, only obesity was related to short sleep (*P* < .05). Among blacks, overweight and obesity were associated with short sleep (*P* < .05). Neither race/nationality nor BMI was significantly associated with long sleep, after stratification by gender. Unadjusted analyses showed patterns similar to adjusted multinomial regression analyses shown in Table 3, with some notable differences. Unlike adjusted analyses, unadjusted analyses showed that (1) among all, being black (*P* < .001) and obesity (*P* < .05) were related to short sleep, (2) among men, being black was associated with short sleep (*P* < .001) while overweight was associated with long sleep (*P* < .05), (3) among women, obesity was associated with short sleep (*P* < .05), and (4) among blacks, overweight was associated with short sleep (*P* < .05).

Among all participants, sleep quality (getting enough sleep to feel rested in the morning in the past 7 days) was worse in blacks (*P* < .05) and Koreans (*P* < .001) than whites, worse in women than men (*P* < .05), and worse in participants with obesity than those who are normal weight (*P* < .05) (Table 4). BMI was no longer a significant predictor of sleep quality after stratification by gender and race/nationality. Among men, sleep quality was poorer in blacks (*P* < .01) and Koreans (*P* < .001) than whites. Among women, it was poorer in Koreans than whites (*P* < .05). Among whites, it was poorer in women than men (*P* < .01). No difference was observed among blacks or Koreans.

**Table 4:** Poisson regression of getting enough sleep in the past 7 days (*N* = 1578)

	All IRR (95% CI)^a^	Men IRR (95% CI)^a^	Women IRR (95% CI)^a^	Whites IRR (95% CI)^a^	Blacks IRR (95% CI)^a^	Koreans IRR (95% CI)^a^
Race/nationality						
Whites	Reference	Reference	Reference	–	–	–
Blacks	0.89 (0.81, 0.98)^*^	0.85 (0.75, 0.96)^**^	0.99 (0.84, 1.15)	–	–	–
Koreans	0.79 (0.73, 0.86)^***^	0.77 (0.70, 0.85)^***^	0.82 (0.70, 0.95)^*^	–	–	–
Gender						
Men	Reference	–	–	Reference	Reference	Reference
Women	0.92 (0.86, 0.99)^*^	–	–	0.79 (0.68, 0.91)^**^	1.00 (0.90, 1.12)	0.91 (0.82, 1.02)
BMI						
Underweight	0.88 (0.71, 1.09)	0.92 (0.62, 1.35)	0.92 (0.70, 1.20)	0.98 (0.48, 1.97)	1.06 (0.67, 1.67)	0.92 (0.68, 1.12)
Normal weight	Reference	Reference	Reference	Reference	Reference	Reference
Overweight	0.96 (0.89, 1.03)	0.95 (0.87, 1.03)	0.95 (0.83, 1.08)	0.92 (0.79, 1.08)	0.99 (0.87, 1.12)	0.97 (0.87, 1.08)
Obesity	0.92 (0.85, 0.99)^*^	0.91 (0.82, 1.01)	0.90 (0.79, 1.03)	0.86 (0.70, 1.06)	0.90 (0.78, 1.04)	0.97 (0.86, 1.09)

Underweight (BMI < 18.5), normal weight (18.5 ≤ BMI < 25.0), overweight (25.0 ≤ BMI < 30.0), and obesity (BMI ≥ 30.0) for whites and blacks. Underweight (BMI < 18.5), normal weight (18.5 ≤ BMI < 23.0), overweight (23.0 ≤ BMI < 25.0), and obesity (BMI ≥ 25.0) for Koreans

^a^Incidence rate ratio (IRR) adjusted for age, current smoking status, days of alcohol use, and depression

*CI* confidence interval

^*^p < .05; ^**^p < .05; ^***^p < .001

**Fig. 1:**
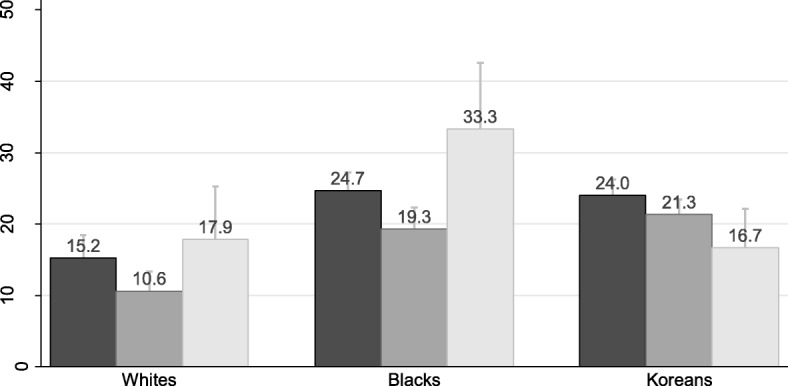
Prevalence of obesity by sleep duration with standard error of the mean. Black bar, short sleep (< 7 h/night). Dark gray bar, normal sleep (7–9 h/night). Light gray bar, long sleep (> 9 h/night)

As shown in Fig. 1, both short and long sleep were significantly associated with obesity in blacks only (*P* < .05).

## Discussion

This study aimed to examine associations of BMI-based weight status with sleep duration and quality among college students in the U.S. and Korea. Moreover, this study tested gender and racial/national differences in the associations. To our knowledge, this study may be the first investigation that examined racial/national differences in the association between weight status and sleep problems among a binational sample of college students.

In this study, overweight and obesity were positively associated with having sleep problems. Specifically, college students with obesity were more likely to have inappropriate sleep duration and poor sleep quality compared to their normal weight counterparts. Previous studies also reported similar associations [53–55]. The association between weight status and sleep problems may be explained by a bidirectional mechanism [15, 16, 56]. Having excess body weight can lead to sleep problems due to disordered breathing [17], while having sleep problems can also contribute to weight gain due to abnormal changes in metabolic and endocrine functions [15, 16]. In another study of college students, however, sleep duration was not significantly associated with weight status, whereas sleep disturbance was significantly associated with increased BMI [12, 18, 33, 34]. Although sleep quality may be a better predictor of sleep-related health outcomes, both sleep duration or quantity and quality are important for well-being [57]. Given a tendency of easy weight gain among college students after starting college life [58, 59], managing uncontrolled weight gain among college students would be supportive to prevent their sleep problems.

Regarding gender differences, there was no specific difference in the association between weight status and sleep problems. Obesity was positively associated with having inappropriately short sleep duration in both men and women, while statistically significant association between weight status and sleep quality was not detected in both genders. Previous studies reported gender differences in overweight/obesity and sleep problems [60–62]. In general, male college students have higher prevalence of overweight/obesity compared to their female counterparts in both the U.S. [60] and Korea [61]. In contrast, female college students are more likely to experience sleep problems than their male counterparts in both countries [62, 63]. Nevertheless, in the association between these two health issues, no difference between men and women existed among this sample of students. Further investigations using a larger sample of college students in both countries may be required to confirm gender differences in the association between excess body weight and sleep problems.

In stratified analyses by college students’ race/nationality, obesity was associated with having inappropriately short sleep duration in only black college students, whereas there was no statistically significant association in white and Korean college students. Similarly, a previous study among a nationally representative sample of U.S. adults reported that only blacks showed a significant association between objectively measured obesity and inappropriately short sleep duration, whereas this association was not statistically significant in whites, Hispanics, and Asians [64]. Generally, black college students have higher prevalence of overweight and obesity than that of white and Asian college students [65]. If the association between obesity and having sleep problems is true, black college students may be the most vulnerable population to sleep problems among college students in the U.S. In fact, a review study documented that blacks have higher prevalence of inappropriately short or long sleep duration and poor sleep quality (less deep and restful sleep), compared to other racial/national groups [66]. Black college students may be in needs of targeted interventions for both weight and sleep management. Additional investigations on comparisons of the association between weight status and sleep problems among multi-national sample of college students may be needed to examine racial/national disparities more accurately.

### Limitations

This study has several limitations. Since this study was a cross-sectional study, cause-and-effect associations cannot be determined. Secondly, as other observational studies, there might be unmeasured confounding factors which affect the study results. For example, influences of common behavioral factors for delayed sleep onset among young people, including caffeine intake and use of electronics late at night [67], were not considered. Thirdly, although objectively measured weight status was used, self-reported sleep duration and quality were used, so this might have affected the study results. Fourthly, when interpreting the data presented in this paper, cautions should be considered because the reliability and validity of NHANES questions used in this study have not been determined. Lastly, this study used a convenience sample of college students which is likely to be biased and have a generalizability issue.

## Conclusions

Despite the limitations, this study provides novel information about the association of weight status with sleep duration and quality among college students in the U.S. and Korea, as well as racial/national differences in the association among three racial/national groups including whites, blacks, and Koreans. Overall, obesity was associated with having inappropriate sleep duration and poor sleep quality among college students in the U.S. and Korea. Although this association was not different by gender, only black students showed significant association between obesity and having inappropriately short sleep duration in stratified analyses. Further investigations using a larger sample of college students in multiple countries may be helpful to identify the populations at a greater risk of overweight/obesity and sleep problems by college students’ gender and race/nationality.

## Data Availability

Data can be made available through the authors.

## Abbreviations

ACHA-NCHA: American College Health Association-National College Health Assessment
ANOVA: Analysis of variance
AOR: Adjusted odds ratio
BMI: Body mass index
CES-D: Center for Epidemiologic Studies Depression Scale
CI: Confidence interval
IRR: Incidence rate ratio
NHANES: National health and nutrition examination survey
NIH: National Institute of Health
SD: Standard deviation
WHO: World Health Organization.
